# Fused Deposition Modeling of Polyamides: Crystallization and Weld Formation

**DOI:** 10.3390/polym12122980

**Published:** 2020-12-14

**Authors:** Andrea Costanzo, Umberto Croce, Roberto Spotorno, Seif Eddine Fenni, Dario Cavallo

**Affiliations:** Department of Chemistry and Industrial Chemistry, University of Genoa, Via Dodecaneso 31, 16146 Genova, Italy; andrea.costanzo@edu.unige.it (A.C.); umberto.croce95@gmail.com (U.C.); roberto.spotorno@unige.it (R.S.); seifeddinefenni@gmail.com (S.E.F.)

**Keywords:** polyamides, 3D printing, crystallization

## Abstract

International newspapers and experts have called 3D printing the industrial revolution of this century. Among all its available variants, the fused deposition modeling (FDM) technique is of greater interest since its application is possible using simple desktop printers. FDM is a complex process, characterized by a large number of parameters that influence the quality and final properties of the product. In particular, in the case of semicrystalline polymers, which afford better mechanical properties than amorphous ones, it is necessary to understand the crystallization kinetics as the processing conditions vary, in order to be able to develop models that allow having a better control over the process and consequently on the final properties of the material. In this work it was proposed to study the crystallization kinetics of two different polyamides used for FDM 3D printing and to link it to the microstructure and properties obtained during FDM. The kinetics are studied both in isothermal and fast cooling conditions, thanks to a home-built device which allows mimicking the quenching experienced during filament deposition. The temperature history of a single filament is then determined by mean of a micro-thermocouple and the final crystallinity of the sample printed in a variety of conditions is assessed by differential scanning calorimetry. It is found that the applied processing conditions always allowed for the achievement of the maximum crystallinity, although in one condition the polyamide mesomorphic phase possibly develops. Despite the degree of crystallinity is not a strong function of printing variables, the weld strength of adjacent layers shows remarkable variations. In particular, a decrease of its value with printing speed is observed, linked to the probable development of molecular anisotropy under the more extreme printing conditions.

## 1. Introduction

3D-printing technology is a layer-by-layer process that is now used in personal and commercial production where prototyping and customization are required. This technique exploits the principle of additive manufacturing (AM) for making three dimensional complex shape solid objects from a digital file. 3D printing allows fabricating products with complex geometries that would be impossible or very challenging to implement with conventional technique [1,2,3,4,5]. The most popular 3D printing technique is the so called fused deposition modelling (FDM): A solid filament is drawn into a printer nozzle, heated to a temperature where the polymer flows readily, then extruded layer-by-layer onto a build plate. When the layer is completed the buildplate is lowered in the z-direction and the next layer is built on top. The fused material exiting the nozzle solidifies upon cooling. One of the negative characteristics of 3D printing products is that they are weaker than those obtained using traditional techniques. In particular, it is observed that the overall strength is much lower when the load is applied in the printing direction with respect to the perpendicular direction. This means that the weak point of the 3D printing products is the contact surface between adjacent layers [6,7]. Seppala et al. used IR thermography to monitor the profile of these regions during printing [8]. The inter-layer welding formation mechanism occurs by diffusion between adjacent chains in different layer, with the eventual formation of entanglements between them [9,10]. In the case of amorphous polymers, the inter-chain diffusion process is opposed by the rapid temperature decrease and ceases when the glass transition temperature is reached [11,12]. Zhou et al., using PC/ABS-based composites, demonstrated how a proper choice of processing conditions might reduce porosity in the manufactured samples, thus allowing to obtain better final mechanical properties [13]. In the case of semi-crystalline polymers, the welding efficiency is instead linked to the quantity of polymer that managed to diffuse before crystallization occurs. FDM is a complex process, characterized by a large number of parameters that influence the quality and final properties of the product. Since the mechanical properties are crucial to guarantee the functionality of the manufactured articles, it is absolutely essential to examine the influence of the process parameters on the mechanical performance [14]. In particular, the use of semicrystalline polymers would guarantee higher mechanical properties than amorphous ones. For this reason, it becomes important to understand how crystallization is influenced by the printing process, trying to define, for each semi-crystalline polymer, a workability window that allows to obtain the best possible final properties. Mechanical properties have a close relationship with the state of aggregation of macromolecular chains [15]: In the crystalline region the chains organize themselves with greater order and are affected by stronger intermolecular forces, causing greater strength and rigidity; macromolecular chains in the amorphous region, on the other hand, prefer to intertwine freely and are easier to disentangle, showing good extensibility. Therefore, it is possible to state that a higher crystallinity of the printed sample leads to a better tensile strength and a higher elastic modulus, while the presence of the amorphous region could greatly increase the elongation at break along the printing direction. In [16] Yang et al. studied the influence of thermal conditions on crystallinity and mechanical properties during the processing of poly(ether-ether-ketone) (PEEK) in 3D printing. PEEK samples were printed by varying the chamber temperature and the nozzle temperature. The results show that the crystallinity can grow from 17% to 31%, as the ambient temperature increases from 25 °C to 200 °C, and the final mechanical properties are strictly related to the printing chamber temperature. Also, nozzle temperature is found to be an influential factor to PEEK’s crystallinity and mechanical properties, because this temperature can affect both the crystal melting process, the crystallization process, the interface between printing lines, and the degradation phenomena of the polymer. It was also verified that by using a high nozzle temperature better adhesion at the interface between the different chains is achieved, allowing the obtaining of a greater product rigidity [17]. Geng et al., conducted a study similar to on poly(phenylene sulfide) (PPS). It was found that non-heat treated 3D printed samples show higher elongation at break and exhibit ductile behavior with plastic deformation. The isothermally crystallized samples, on the other hand, do not show any evident plastic deformation, since as the load increases, rapid breakage is observed after reaching maximum stress. These results indicate that a brittle–ductile transition occurred when the chamber was heated above the crystallization temperature. The tensile strength and the elastic modulus increase with increasing the severity of the heat treatment undergone by the samples. This phenomenon is related to the crystallinity of the material, which also increases according to the heat treatment. It is worth noting that the mechanical properties also depend on the macroscopic structure obtained following the printing process; it has been observed that with an increase in the temperature of the heat treatment, the gap between adjacent filaments is reduced and diffusion between the interlayer increases. [18] Vaes et al. monitored the effect of process parameters, including printing speed, build plate and liquefier temperature on the thermal history of two random PA 6/66 copolymers of different molecular weights. By monitoring the layer temperatures during printing via IR thermography, temperature profiles as a function of time were obtained, which were subsequently simulated in a rapid scanning chip calorimetry (FSC) device. The FSC has been shown to be able to mimic the high heating and cooling rates, which are typically experienced by the extruded polymer during the FDM process and cannot be achieved with conventional DSC. In this way it was possible to monitor the progress of crystallinity in a layer during printing and the influence of each of the printing parameters. It has been found that both the nozzle temperature and the printing speed greatly affect the total crystallinity reached by the sample. Furthermore, it has been found that buildplate temperature had a significant impact on the temperature experienced by the printed layers. In fact, a rapid decrease in temperature experienced by the printed layers is observed as the temperature of the plate decreases. The layers deposited closer to the buildplate are also more crystalline due to annealing above the glass transition temperature of the material through conduction heating from the build plate. [19] Lily et al. studied the effects of the processing conditions on the crystallization kinetics of polycaprolactone by combining a 3D printer with an online Raman spectroscopy measurement system. In this work we propose the study of the printability of two polyamides, a Nylon6 homopolymer (Novamid ID 1070) and a random copolymer consisting of Nylon6 and Nylon6-6 monomers (Novamid ID 1030). The first part of the work involved the characterization of the crystallization kinetics of the two materials in isothermal and fast cooling conditions. Once the crystallization kinetics of the two materials were defined, we focused on studying the thermal history undergone by the polymers during the deposition process using a micro-thermocouple. The aim was to measure the temperature variation in the first layer of deposited material and to verify possible variations in the cooling rate depending on the selected printing conditions. A similar approach has already been proposed by Srinivas et al., for printed polylactides [20]. Finally, the work involved measuring the weld strength for the two materials for a wide range of printing conditions, verifying for which combinations of parameters the best mechanical response can be obtained.

## 2. Materials and Methods

**Figure 1:**
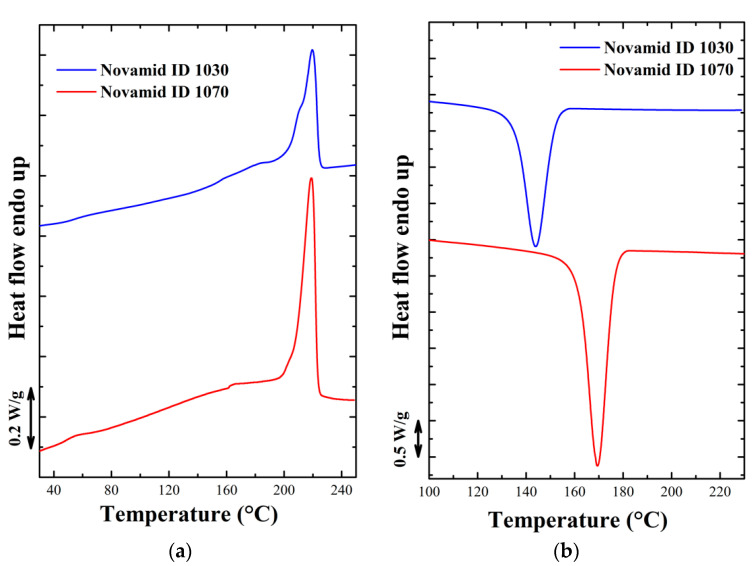
(**a**) First heating ramp and (**b**) cooling ramp at 10 °C/min for the two polyamides obtained by DSC calorimetric analysis.

The materials chosen for this work are marketed by DSM under the names Novamid ID 1070 and ID 1030, which are a polyamide-6 homopolymer and a polyamide6-6,6 copolymer respectively [21]. As can be observed from the standard calorimetric analysis (Figure 1a,b), carried out using a DSC 250 from TA Instruments (New Castle, DE, USA), these are two semi-crystalline polymers which differ both in melting temperature and degree of crystallinity.

The non-isothermal characterization was performed with a purposely-developed quenching device [22,23]. The sample, once prepared in the form of a thin film with a thermocouple inside, is fixed on a vertical support. In this position the sample is heated to above its melting temperature by means of a heating gun, and subsequently cooled at high speeds using compressed air flows from both sides through two flexible pipes. The cooling speed is varied by adjusting the air flow. The specimen for testing the weld strength were prepared by means of an Intamsys Funmat HT 3D printer (Shanghai, China), equipped with a nozzle of 0.4 mm diameter. To protect the feedstocks from the effects of humidity, before printing the samples, the spools were dried in an oven for 24 h at 80 °C. To simplify as much as possible the preparation of the samples in view of the subsequent measurements, the chosen printed geometry, analogous to that in [24], is that of a free-standing square tube (4 cm × 4 cm × 4 cm in size) consisting of a single-filament stack with layer height of 0.4 mm. This means that, within each layer, the nozzle moves along a square path and extrudes a single polymer filament. In this way problems of inter-layer voids are eliminated, and the mechanical test probes a single weld surface only. Sample with the described geometry was designed with the software Tinkercad and subsequently converted into an STL format file for printing. The software (Ultimaker, Utrecht, The Netherlands) Cura was used to generate G-code and to set up the processing conditions through a slicing process on the original file [25]. Specimens were printed exploring different processing conditions, namely by varying nozzle temperature and print speed, while keeping the bed and chamber temperatures constant at 60 °C and 50 °C, respectively. Specimens were subjected to a tensile test (ASTMD1938) [26] to determine weld strength as a function of printing conditions. The printed shapes (square tubes) were first cut along the four side walls using a scissor. Then, three rectangular-shaped tensile specimens for each print condition were punched out from each side wall using a pneumatic press. The layer deposition direction was oriented at an angle of 90° with respect to the cutting direction. The tested samples have the following dimensions: 40 mm of height, 13 mm of width and roughly 0.45 mm thickness. Rectangular-shaped (single-filament wall) specimens are chosen, rather than a traditional dog-bone shape, due to geometrical constraints imposed by the print size and the available blade to punch-out the sample.

## 3. Results and Discussion

### 3.1. Thermal Analysis

#### 3.1.1. Isothermal Characterization

**Figure 2:**
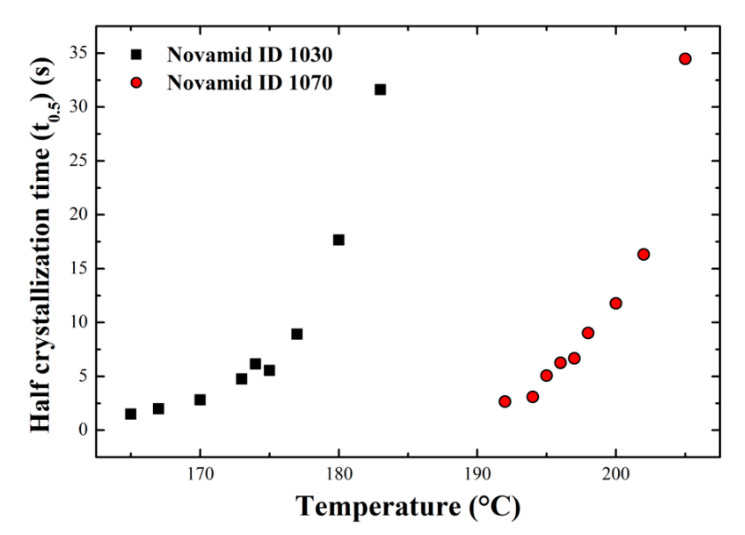
Half crystallization time for the two polyamides as a function of the crystallization temperature.

**Figure A1:**
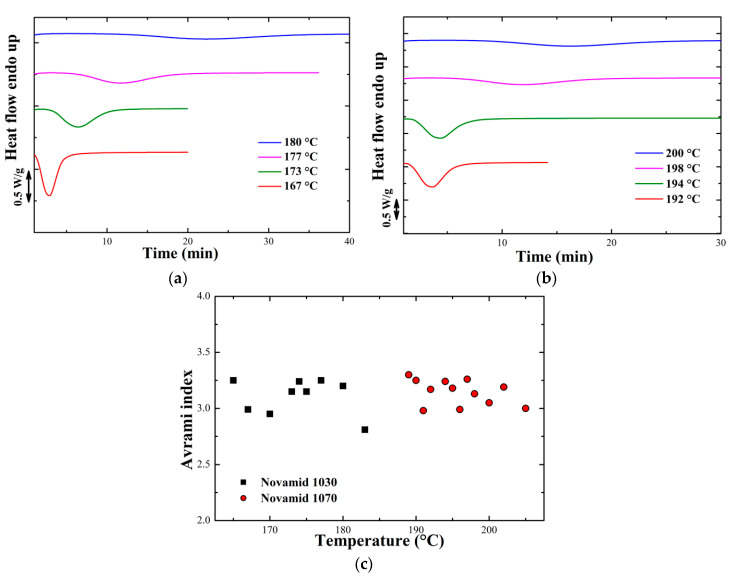
Examples of isothermal crystallization curves for (**a**) Novamid ID 1030 and (**b**) Novamid ID 1070; (**c**) Avrami index of the two polyamides.

A characterization of the materials’ crystallization behavior has been performed according to the Avrami model [27]. Avrami’s equation allows to describe, at constant temperature, the changes in the states of aggregation of materials and is particularly useful in the context of polymeric materials to obtain important information relating to their crystallization kinetics. Once the onset points corresponding to the start of crystallization in a standard heating-cooling cycle were individuated, a series of isotherms at gradually increasing temperatures was performed on the two polyamides, starting from about 15 °C above the temperature of crystallization onset. Some of the isothermal crystallization curves for the two materials are shown in the Appendix A (Figure A1a,b). By means of a plug-in for the OriginPro software, it was possible to analyze the isothermal crystallization curves in order to obtain the half-crystallization times (t_0.5_) for the two materials [28]. The obtained values are reported in Figure 2. As expected, in the case of the homopolymer, a shift to higher temperatures is required to achieve the same crystallization rate of the copolymer, indicating a largely different crystallization kinetics for the two polyamides. The difference in the undercooling at the same rate for the two materials are in the order of 20 °C. Thanks to the same plug-in it was also possible to calculate the Avrami index for the two materials, reported in Appendix A (Figure A1c). An Avrami exponent of 3 is obtained, suggesting classical spherulitic crystallization on heterogeneous nuclei.

#### 3.1.2. Non Isothermal Characterization

**Figure 3:**
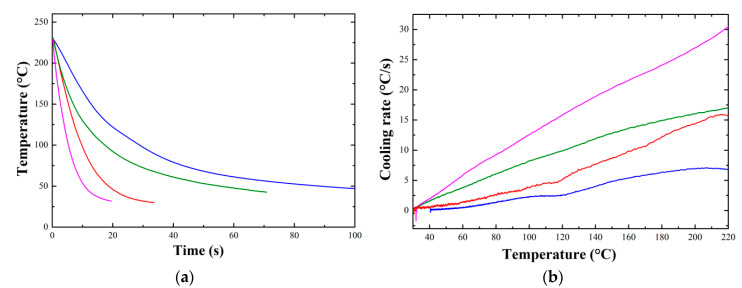
(**a**) Cooling curves obtained for different air flows and (**b**) corresponding cooling rates for Novamid ID 1030 samples.

The characterization of the crystallization kinetics in fast cooling conditions was carried out using a controlled air flow, thanks to which it was possible to obtain cooling rates of increasing intensity. Samples for the two materials were prepared in the form of thin films, heated above their respective melting temperatures and subsequently cooled. The thermal history of the samples was measured using a type K thermocouple inserted into the thin film and connected to a National Instrument acquisition unit. Figure 3a shows the cooling curves for the Novamid ID 1030 for different air flows, while Figure 3b shows the corresponding cooling rates obtained by numerical derivation of the temperature profiles. Appendix B shows the same curves for the Novamid ID 1070. It can be seen that the temperature decreases exponentially to the target value (room temperature), and that the decrease is somehow perturbed at the lower cooling rate by the heat released by crystallization, which provokes a temperature plateau. Cooling rates are linearly dependent on temperature when no phase transition occurs. As such, the crystallization process can be detected at the lowest cooling rate as a peak in the derivative curve (Figure 3b). We note that cooling rates of few to tens °C/s are obtained, exceeding those commonly achievable by DSC, but in the range of those expected for FDM 3D printing [8]. Some of the quenched samples were subsequently subjected to a heating ramp at 10 °C/min at the DSC in order to highlight possible differences of crystallinity with cooling conditions.

**Figure 4:**
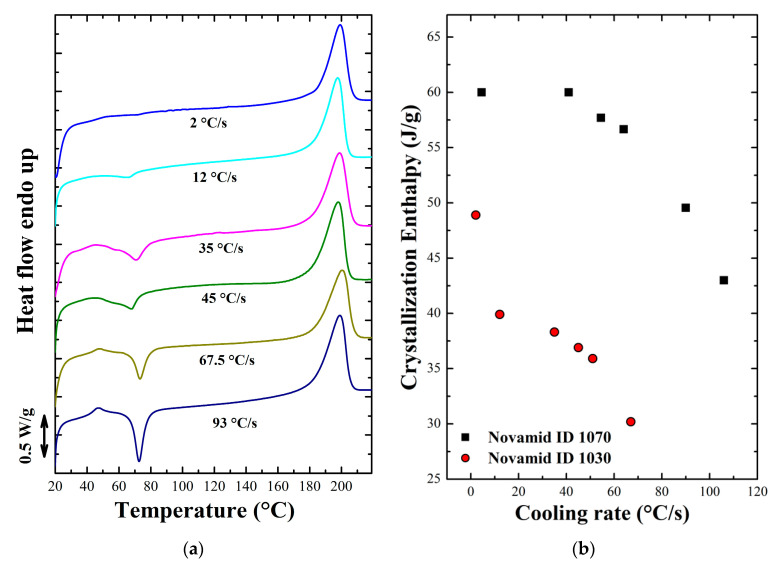
(**a**) DSC heating ramps obtained for Novamid ID 1030 samples subjected to the indicated different cooling rates. Cooling rates are estimated at 180 °C. (**b**) Crystallization enthalpy for samples of the two polyamides as the average cooling rate varies.

In the case of the Novamid ID 1030, the Figure 4a shows that, as the cooling rate increases, the area of the cold crystallization peak increases, correlated to the heat released during the crystallization process in the heating ramp. This means that, with faster cooling, the material will have a gradually lower initial degree of crystallinity before the DSC heating ramp. The enthalpy of crystallization during the fast cooling can thus be derived by subtracting the cold crystallization enthalpy from the melting endotherm. Therefore, the initial degree of crystallinity shows a decrease as a function of the cooling rate for both polyamides as displayed in Figure 4b. The crystallinity decreases of about one third with an increase of cooling rate from few to hundreds °C/s. The same heating ramps for the other polyamide are shown in Appendix C. In fact, at high cooling rates, the time allowed for the polymer chains during solidification to adjust into ordered structures is shorter, thus preventing the material from reaching the maximum achievable degree of crystallinity. The crystallization kinetics of polyamide 6,6 in non-isothermal conditions had already been studied by Rhoades et al., who found a similar trend to the present case [29].

The large difference in crystallizability of the two materials, as evidenced by isothermal and non-isothermal measurements, might result in different welding performance during printing. In principle, the polyamide copolymer crystallizes at lower temperature during cooling which give rise to longer welding times and potentially more efficient weld formation. However, the different nozzle temperatures for the two polymers complicate the above reasoning. On the other hand, the different crystallinity between homo- and copolymer can influence the ultimate achievable tensile strength, even if a similar bond between the layers is achieved during cooling the deposited filaments.

#### 3.1.3. In-Situ Temperature Measurements

**Figure 5:**
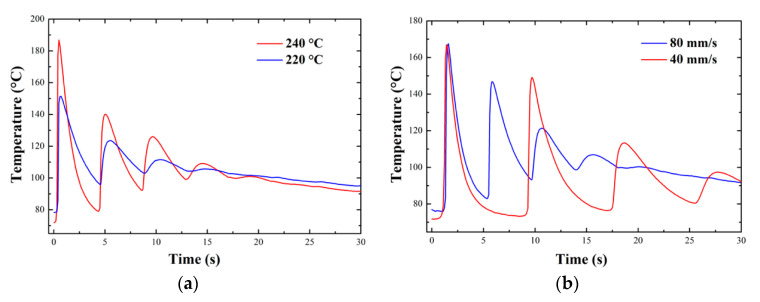
Thermal profiles of the first layer of Novamid ID 1030 deposited on the plate for different printing conditions at the same deposition speed (80 mm/s) (**a**) and the same nozzle temperature (230 °C) (**b**).

Having characterized the crystallization behavior of the materials under isothermal and non-isothermal processing conditions, the work focused on determining the thermal history of the material during the deposition process. Several previous works have already proposed set-ups able to measure the thermal profile of the material during the deposition process, for example by IR thermography or fine thermocouples [19,30]. The printed geometry is the same as previously described and which was also used for the weld strength measurements described further on. Using a micro-thermocouple placed on the printing plate in correspondence with the deposition area, it was possible to measure the thermal profile of the first layer following its deposition and that of the subsequent layers above it. Figure 5 shows the thermal profile for Novamid ID 1030 at different printing speed and at different nozzle temperatures respectively. Note that the maximum temperature measured by the thermocouple does not correspond to that set in the nozzle, probably due to the very short contact time between the two. Each temperature peak corresponds to a passage of the nozzle over the area of the plate where the thermocouple has been placed. The printing process was allowed to proceed until the deposition of successive upper layers led to temperature peaks of appreciable intensity. For both polyamides the thermal profile was measured at three printing speeds (20, 60, and 120 mm/s) and for three different nozzle temperatures for the two materials (220, 230, and 240 °C for the Novamid ID 1030 and 255, 265, and 270 °C for the Novamid ID 1070). Figure 5 shows some examples of thermal history for Novamid ID 1030 respectively at constant deposition temperature (a) and constant deposition rate (b). It can be seen how different nozzle temperatures lead to an evident variation of the maximum amplitude, while different printing speeds lead to variation of the modulation period of the temperature/time curves. Appendix D provides similar examples for the Novamid ID 1070. It can also be observed that the minimum temperature reached after subsequent depositions increases, reasonably due to the increasing number of underlying hot layers deposited which therefore slow down the dissipation of heat during cooling. Moreover, it is worth to note that the average temperature in the long time keeps definitely higher than the glass transition. As such, crystallization is expected to proceed until saturation, given the recorded temperature history of the deposited filament.

**Figure 6:**
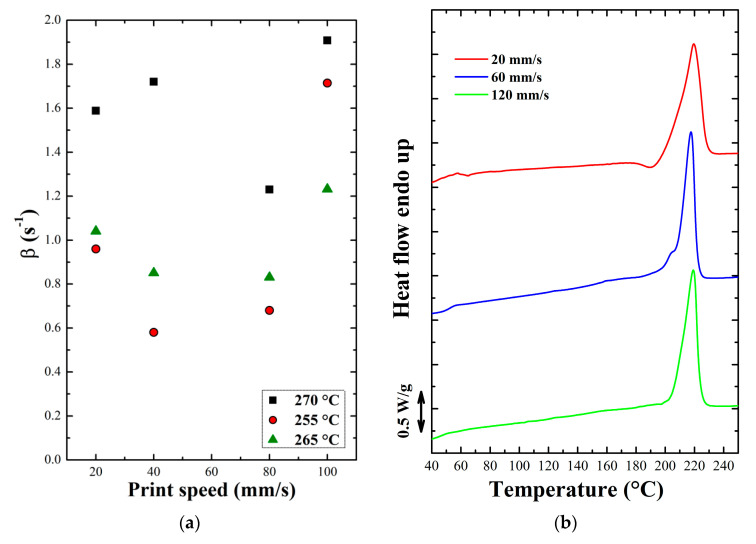
(**a**) Average values of parameter β calculated for the different printing conditions and (**b**) DSC ramps in heating at 10 °C/min for samples printed at 255 °C of Novamid ID 1070.

**Figure A3:**
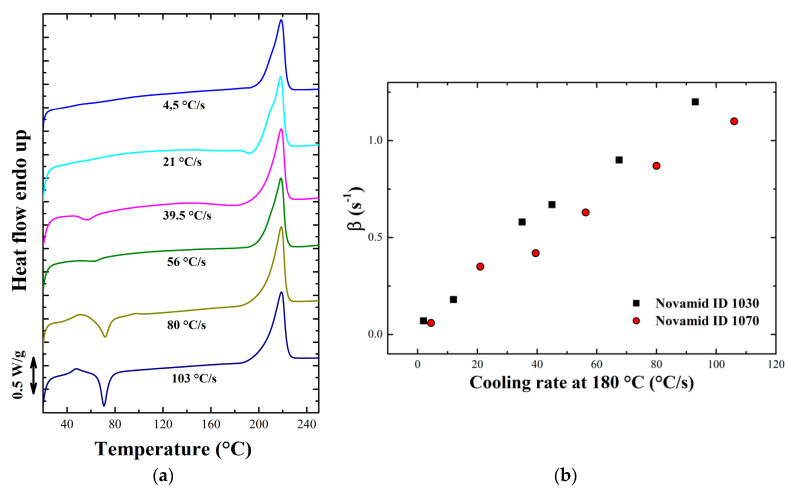
(**a**) DSC heating ramps obtained for Novamid ID 1070 samples subjected to different cooling rates; (**b**) Parameter β estimated for the two polyamides as a function of the cooling rate calculated at 180 °C.

To characterize the thermal history corresponding to the different printing conditions the peak relative to the first passage of the nozzle on the thermocouple was considered, representing the deposition of the first layer. By analyzing the cooling curves in the different cases and obtaining the respective cooling rates, it is possible to estimate a parameter (betha), which represents the reciprocal of the time constant for the exponential cooling of the single filament [31]. The betha parameter (β) was calculated for all the analyzed printing conditions and the results in the case of Novamid ID 1070 are shown in Figure 6a. The trend of parameter ß found for the various process conditions highlights the fact that the deposition speed does not meaningfully influence it. In fact, the average β value remains constant as the printing speed increases but changes, shifting to higher values, with increasing the nozzle temperature. The samples for which thermal history was measured were subsequently subjected to a heating ramp at 10 °C/min in the DSC. Figure 6b shows the heat flow related to the heating ramps for the Novamid ID 1030 samples printed at 20, 60, and 120 mm/s at the nozzle temperature of 230 °C. The heat flow curves indicate, for all printing conditions, the achievement of the maximum degree of crystallinity during cooling, as judged by the absence of cold crystallization. In fact, by comparing the values of β for the printed sample with those obtained for quenched samples, it is possible to derive that a β of around 1.2 s^−1^ correspond to a cooling rate, calculated at 180 °C, of about 100 °C/s (see Figure A3b). Such high cooling rates, as previously seen, should have led to the appearance of cold crystallization peaks during subsequent heating. However, this does not happen because, as shown by the thermal history of the layer during printing, the polymers remain above the glass transition temperature (about 60 °C) for most of the deposition process, thus giving the possibility to the material to fully crystallize. Therefore, in the range of cooling rates corresponding to the printing conditions, the enthalpy of crystallization corresponds to the maximum achievable by the material. The achievement of the maximum degree of crystallinity in all the cases may also be due to the effect induced by the flow to which the materials are subjected during the deposition process, which can accelerate the crystallization kinetics. Being able to cool in relatively mild conditions during the printing process, the materials do not exhibit the characteristic peak of cold crystallization when subjected to a subsequent heating ramp. A noteworthy exception is that of the sample printed at 20 mm/s which shows a distinct exothermic signal before melting, possibly indicating a reorganization phenomenon of the crystals. For that sample the width of the melting endotherm is also broader than the rest of the conditions, suggesting that a different microstructure might be present. Similar cold-ordering event are observed for samples of polyamide-6 crystallized at large undercooling into a mesomorphic structure [22].

#### 3.1.4. Weld Strength Measurements

Next, results on the adhesion strength between adjacent layers and how this vary with processing conditions are presented. As already demonstrated, the processing parameters can be a fundamental factor in determining a better adhesion between adjacent layers [32], sometimes allowing the use of the products even in extreme temperature conditions [33]. Table 1 summarizes the ranges of variation of the printing conditions, for which the mechanical properties were analyzed.

**Figure 7:**
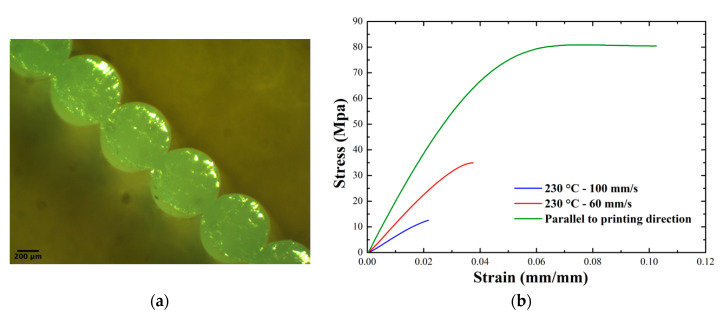
(**a**) An example of micrograph of the deposited layers for a sample printed at 230 °C and at a speed of 60 mm/s; (**b**) examples of stress–strain curves obtained from tensile tests on Novamid ID 1030 samples.

All samples were printed maintaining the buildplate and printer chamber temperatures at 80 °C and 50 °C, respectively. After printing the cubes and cutting out the faces from which the specimens for mechanical tests were obtained, in order to correctly assess the stress it was necessary to measure the contact surface between adjacent layers using a stereoscope. An example photomicrograph of the weld width measurement is shown in Figure 7a. Tensile tests were carried out by setting a clamp separation speed of 6 mm/min. Some examples of the stress–strain curves obtained for Novamid ID 1030 are shown in Figure 7b. Appendix D provides similar examples for the Novamid ID 1070. In the proposed examples, it is generally noted that the tensile strength and deformation at break are lower the more extreme the printing conditions of the materials are i.e., when one is far from the typical processing temperature of the material and at a deposition rate too high or low. These stress–strain curves show how the printing conditions strongly influence the final mechanical properties, in particular the stress at break, and how this value is different when the load is applied perpendicular or parallel to the printing direction (Figure 7b). For both materials, the trend can be traced back to a brittle breakage, therefore preceding the yielding phase, apart from the case indicated as “parallel to the printing direction”, in which yielding and therefore plastic deformation occur. The large difference between loading in the parallel or transverse direction indicate a meaningful anisotropy of the printed object.

**Figure 8:**
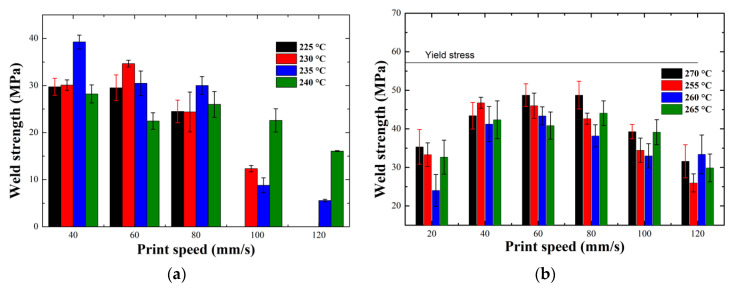
Trend of the weld strength as a function of the different printing conditions for Novamid ID 1030 (**a**) and Novamid ID 1070 (**b**). The horizontal line in figure **b** corresponds to the average yield stress values obtained for the two materials in the case of measurements parallel to the printing direction.

Figure 8 shows the weld strength values for different nozzle temperatures for Novamid ID 1030 (a) and Novamid ID 1070 (b), as the printing speed varies. The horizontal lines in the graphs correspond to the average yield stress values obtained for the two materials in the case of measurements parallel to the printing direction. All measured values, remain lower than those of bulk stress at break, respectively found at 79 and 89 MPa. It should be noted that, in the case of the Novamid ID 1030, there are no data points related to some printing parameters, because of the impossibility in printing the specimen under those conditions. In the case of the copolymer the change in weld strength recalls the behavior already found in amorphous polymers, i.e., the weld strength decreases monotonically with increasing printing speed. In particular, for high printing speeds the drop in weld strength can be associated with the molecular orientation, due to the high flow experienced by the macromolecules (Figure 8a) [34]. If subjected to high shear stresses it is in fact possible to orient the polymer chains in a preferential direction of space. The result of this mechanism is the generation of an anisotropic molecular network, resulting in a decreased density of entanglements in a direction perpendicular to the printing one. In fact, a decreasing trend of weld strength with increasing printing speed had already been found in a previous work, where an amorphous material was used and the presence of molecular orientation at the interfaces between the layers was demonstrated by birefringence measurements [35].

The situation is different in the case of the homopolymer, where the data trend displays a maximum, with the optimum located at intermediate printing speeds with respect to the analyzed range (Figure 8b). It is interesting to note that an optimum value of tensile strength as a function of printing parameters is also achieved in multiple-layer structures, due to a balance between deposition of the filament and bond formation [13]. The poor mechanical properties at the interface for low deposition rates could be due to the peculiarity of the deposition process. We recall in fact that the final DSC heating scan of the Novamid 1070 sample printed at 20 mm/s presents reorganization and broad melting phenomena, indicative of less stable crystals, possibly pertaining to the mesophase [29,36]. We thus propose that the original crystals for the sample printed at 20 mm/s are in mesophase and possess lower mechanical strength. For what concerns the effect of temperature, previous work on amorphous PLA showed that there was a shift to greater values of weld strength as the temperature of the nozzle increases [37]. Instead, for the two polymers considered in this study this process variable does not seem to play a fundamental role for crystallization and final mechanical properties of the printed specimen. This suggest that the welding quality is not limited by the diffusion for this system, being the nozzle temperature always sufficiently high to allow formation of entanglement formation at the interface, unless orientation start to be a dominant factor. In general, the homopolymer Novamid ID 1070 is more resistant to traction than the copolymer Novamid ID 1030.

## 4. Conclusions

3D printing technology has been taking over in recent years, as it allows to create objects with geometries that would not be achievable using other traditional techniques. Although this technology is already widely in use, the number of materials currently usable is still quite limited. The reason for this is that some specific criteria are still missing to determine printability for a given polymer. Although semi-crystalline polymers are characterized by significantly better mechanical properties and thermal stability than their amorphous counterparts, their use as a raw material for 3D printing technology still poses some major challenges. In this regard, it is necessary to fully understand the development of the crystallinity of the printed parts during the production process, in order to control the strength of the weld between the various layers and the final deformation of the products. In this work an approach was proposed to study the influence of crystallization during printing on the final properties, by selecting two polyamides with largely different crystallizability. In particular it was clearly assessed that the polyamide-6 homopolymer crystallizes at higher temperatures or in shorter times than the polyamide 6/6,6 copolymer, as expected from the molecular structure. The application of fast cooling protocols by means of a home-built device revealed a decrease in crystallinity as the cooling rate applied increases for both materials. By analyzing the printed parts via DSC it is finally verified that during the printing process, for both materials, the maximum possible degree of crystallinity is reached. However, low printing speed for the homopolymer probably lead to the development of the mesomorphic modification. A direct measurement of the temperature profile of the filament during the printing process was attempted, and it was concluded that the first filament, after the fourth layer, is no longer affected by the heat generated by the deposition of the subsequent layers, as its temperature remains almost constant as the printing process continues. This temperature is higher than the glass transition temperature of the polymer, which is why, at the end of printing, the polymer was able to reach the maximum degree of developable crystallinity. In the tensile strength or weld strength tests for the homopolymer, a “bell-shaped” trend was obtained, indicating that the best tensile properties are obtained in samples printed at intermediate printing speeds. The maximum loads are in any case lower than the yield strength so no plastic deformation of the material occurs at the interface between the two layers. In the copolymer, on the other hand, the best tensile strength was found at low printing speeds with an important decrease at higher speeds. This fact can be explained by assuming that at high speeds a meaningful degree of molecular orientation is obtained in the transverse direction with respect to the loading. This study represents a first approach in developing deeper knowledge regarding the behavior of semicrystalline polymeric materials in the 3D printing process.

## Figures and Tables

**Table 1:** Adopted processing parameters.

Parameter	Novamid ID 1030	Novamid ID 1070
Nozzle temperature	220–240 °C	255–270 °C
Print speed	20–120 mm/S	20–120 mm/s
Buildplate temperature	80 °C	80 °C
Chamber temperature	50 °C	50 °C

**Figure A2:**
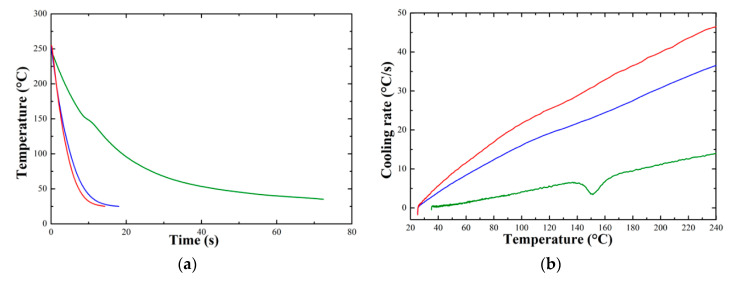
(**a**) Cooling curves obtained at different air flows and (**b**) corresponding cooling rates for Novamid ID 1070 samples.

**Figure A4:**
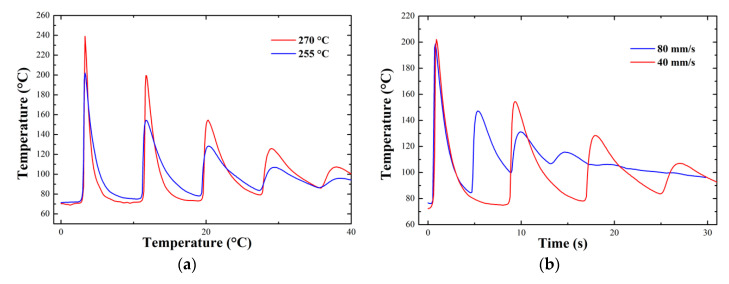
Thermal profiles of the first layer of Novamid ID 1070 deposited on the plate for different printing conditions at the same deposition speed (40 mm/s) (**a**) and the same nozzle temperature (255 °C) (**b**) respectively.

**Figure A5:**
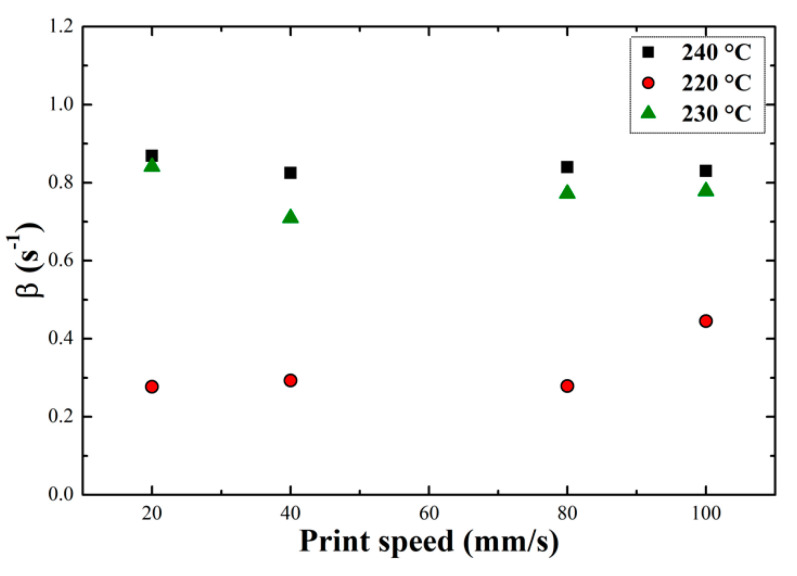
Average values of parameter β calculated for the different printing conditions for Novamid ID 1030 samples.

**Figure A6:**
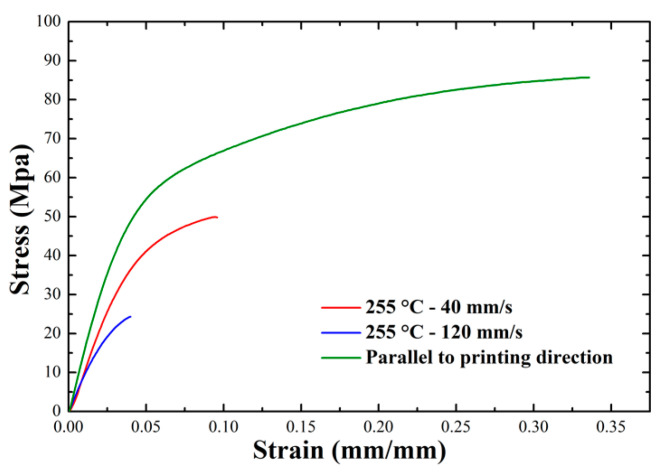
Examples of stress–strain curves obtained from tensile tests on Novamid ID 1070 samples.

## Appendix A. Isothermal Analysis

Figure A1a,b shows some of the isothermal crystallization curves acquired for the two materials. All the temperatures selected for the two polyamides belong to a range positioned at least 15° C above the respective non-isothermal crystallization temperature. Figure A1c shows the Avrami index calculated thanks to the aforementioned Origin plug-in for the two materials. In both cases there is an average value of about 3 for all temperatures, indicating a three-dimensional growth of the crystals.

## Appendix B. Non Isothermal Analysis

Figure A2a shows the cooling curves for the Novamid ID 1030 at room temperature and for different air flows while Figure A2b shows the cooling rates for the same previous conditions.

## Appendix C. DSC Quenched Samples

In the case of the Novamid ID 1030, Figure A3 shows that as the cooling rate increases, the area of the cold crystallization peak increases, correlated to the heat released during the crystallization process in the heating ramp. On the right is shown that parameter β linearly correlates with cooling rate measured at 180 °C.

## Appendix D. In-Situ Temperature Measurements

Figure A4 shows the thermal profiles of the first layer of Novamid ID 1070 deposited on the plate for different printing conditions at the same deposition speed (40 mm/s) (a) and the same nozzle temperature (255 °C) (b) respectively.

## Appendix E. Betha Novamid ID 1070

Figure A5 shows parameter β calculated for the different printing conditions for Novamid ID 1030 samples.

## Appendix F. Weld Strength Tests

Figure A6 shows some examples of the stress–strain curves obtained for Novamid ID 1070.

## References

[1] Gold A., Strong S.R., Turner B.N. (2014). A review of melt extrusion additive manufacturing, processes: I. Process design and modelling. Rap. Prot. J..

[2] Ligon S.C., Liska R., Stampfl J., Gurr M., Mülhaupt R. (2017). Polymers for 3D Printing and Customized Additive Manufacturing. Chem. Rev..

[3] Wendel B., Rietzel D., Kühnlein F., Feulner R., Hülder G., Schmachtenberg E. (2008). Additive Processing of Polymers. Macromol. Mater. Eng..

[4] Kreiger M., Pearce J.M. (2013). Environmental Life Cycle Analysis of Distributed Three-Dimensional Printing and Conventional Manufacturing of Polymer Products. ACS Sustain. Chem. Eng..

[5] Bikas H., Stavropoulos P., Chryssolouris G. (2016). Additive manufacturing methods and modelling approaches: A critical review. Int. J. Adv. Manuf. Technol..

[6] Ahn S., Montero M., Odell D., Roundy S., Wright P.K. (2002). Anisotropic material properties of fused deposition modeling ABS. Rapid Prototyp. J..

[7] Srinivas V., van Hooy-Corstjens C.S., Rastogi S., Harings J.A.W. (2020). Promotion of molecular diffusion and/or crystallization in fused deposition modeled poly(lactide) welds. Polymer.

[8] Seppala J.E., Migler K.B. (2016). Infrared thermography of welding zones produced by polymer extrusion additive manufacturing. Addit. Manuf..

[9] Bellehumeur C., Li L., Sun Q., Gu P. (2004). Modeling of Bond Formation Between Polymer Filaments in the Fused Deposition Modeling Process. J. Manuf. Process..

[10] Croccolo D., De Agostinis M., Olmi G. (2013). Experimental characterization and analytical modelling of the mechanical behaviour of fused deposition processed parts made of ABS-M30. Comput. Mater. Sci..

[11] Kausch H.-H. (1987). Polymers/Properties and Applications.

[12] Seppala J.E., Han S.H., Hillgartner K.E., Davis C.S., Migler K.B. (2017). Weld formation during material extrusion additive manufacturing. Soft Matter.

[13] Zhou Y.-G., Zou J.-R., Wu H.-H., Xu B.-P. (2020). Balance between bonding and deposition during fused deposition modeling of polycarbonate and acrylonitrile-butadiene-styrene composites. Polym. Compos..

[14] Chacón J.M., Caminero M.A., García-Plaza E., Núñez P.J. (2017). Additive manufacturing of PLA structures using fused deposition modelling: Effect of process parameters on mechanical properties and their optimal selection. Mater. Des..

[15] Mark J.E. (2007). Physical Properties of Polymers Handbook.

[16] Yang C., Tian X., Li D., Cao Y., Zhao F., Shi C. (2017). Influence of thermal processing conditions in 3D printing on the crystallinity and mechanical properties of PEEK material. J. Mater. Process. Technol..

[17] Geng P., Zhao J., Wu W., Wang Y., Wang B., Wang S., Li G. (2018). Effect of Thermal Processing and Heat Treatment Condition on 3D Printing PPS Properties. Polymer.

[18] Vaes D., Coppens M., Goderis B., Zoetelief W., Van Puyvelde P. (2019). Assessment of Crystallinity Development during Fused Filament Fabrication through Fast Scanning Chip Calorimetry. Appl. Sci..

[19] Northcutt L.A., Orski S.V., Migler K.B., Kotula A.P. (2018). Effect of processing conditions on crystallization kinetics during materials extrusion additive manufacturing. Polymer.

[20] Srinivas V., Van Hooy-Corstjens C.S., Harings J.A. (2018). Correlating molecular and crystallization dynamics to macroscopic fusion and thermodynamic stability in fused deposition modeling; a model study on polylactides. Polymer.

[21] (2019). DSM develops new 3D printing materials. Reinf. Plast..

[22] Cavallo D., Portale G., Balzano L., Azzurri F., Bras W., Peters G.W., Alfonso G.C. (2010). Real-Time WAXD Detection of Mesophase Development during Quenching of Propene/Ethylene Copolymers. Macromolecules.

[23] Cavallo D., Gardella L., Alfonso G.C., Portale G., Balzano L., Androsch R. (2011). Effect of cooling rate on the crystal/mesophase polymorphism of polyamide 6. Colloid Polym. Sci..

[24] Jin M., Giesa R., Neuber C., Schmidt H. (2018). Filament Materials Screening for FDM 3D Printing by Means of Injection-Molded Short Rods. Macromol. Mater. Eng..

[25] Cheng G.Z., Estepar R.S.J., Folch E., Onieva J., Gangadharan S., Majid A. (2016). Three-dimensional Printing and 3D Slicer: Powerful Tools in Understanding and Treating Structural Lung Disease. Chest.

[26] Massey L.K. (2004). Plastics Design Library, Film Properties of Plastics and Elastomers.

[27] Hubbes S.-S., Danzl W., Foerst P. (2018). Crystallization kinetics of palm oil of different geographic origins and blends thereof by the application of the Avrami model. LWT.

[28] Lorenzo A.T., Arnal M.L., Albuerne J., Müller A.J. (2007). DSC isothermal polymer crystallization kinetics measurements and the use of the Avrami equation to fit the data: Guidelines to avoid common problems. Polym. Test..

[29] Rhoades A.M., Williams J.L., Androsch R. (2015). Crystallization kinetics of polyamide 66 at processing-relevant cooling conditions and high supercooling. Thermochim. Acta.

[30] Sun Q., Rizvi G.M., Bellehumeur C.T., Gu P. (2008). Effect of processing conditions on the bonding quality of FDM polymer filaments. Rapid Prototyp. J..

[31] McIlroy C., Seppala J.E., Kotula A.P. (2019). Combining Modeling and Measurements To Predict Crystal Morphology in Material Extrusion. ACS Symp. Ser..

[32] Zhang X., Wang J. (2020). Controllable interfacial adhesion behaviors of polymer-on-polymer surfaces during fused deposition modeling 3D printing process. Chem. Phys. Lett..

[33] Cruz P., Shoemake E.D., Adam P., Leachman J. (2015). Tensile strengths of polyamide based 3D printed polymers in liquid nitrogen. IOP Conf. Series: Mater. Sci. Eng..

[34] Ghodbane S.A., Murthy N.S., Dunn M.G., Kohn J. (2019). Achieving molecular orientation in thermally extruded 3D printed objects. Biofabrication.

[35] Costanzo A., Spotorno R., Candal M.V., Fernández M.M., Müller A.J., Graham R.S., Cavallo D., McIlroy C. (2020). Residual alignment and its effect on weld strength in material-extrusion 3D-printing of polylactic acid. Addit. Manuf..

[36] Mileva D., Kolesov I., Androsch R. (2012). Morphology of cold-crystallized polyamide 6. Colloid Polym. Sci..

[37] Levenhagen N.P., Dadmun M. (2017). Bimodal molecular weight samples improve the isotropy of 3D printed polymeric samples. Polymer.

